# Stunning Popularity of LARCs With Good Access and Quality: A Major Opportunity to Meet Family Planning Needs

**DOI:** 10.9745/GHSP-D-15-00044

**Published:** 2015-03-02

## Abstract

Given true choice, a very high proportion of women, perhaps most, would select one of the long-acting reversible contraceptives (LARCs)—implants or IUDs—for contraception. If implemented on a wide scale, it would not only drastically alter the current method mix but also serve client needs much better and prevent unintended pregnancy more successfully.

***See related articles by*** ***Curry***, ***Part I******;*** ***Curry***, ***Part II******; and*** ***Ross*****.**

There is no such thing as a precisely optimal contraceptive method mix. Individual needs and preferences vary widely, as do cultures and stage of reproductive life. But what might the method mix look like if women and couples were really provided with good choice from among a full range of methods? The Contraceptive CHOICE project in the United States found that a majority of 2,500 young women chose to use one of the long-acting reversible contraceptive (LARC) methods of either implants or intrauterine devices (IUDs).^1^

Now also witness the striking findings from a very different setting—CARE's very large family planning intervention in 5 crisis-affected countries, reported in this issue of GHSP.^2^^,^^3^ In this well-executed substantial program, in which more than 52,000 women who were given a wide choice of methods started using a modern method, overall a *remarkably high 61%* of them selected LARCs (Figure). To appreciate fully this high proportion, we can compare it to the extensive global data on method mix compiled by Ross et al., also in this issue of GHSP.^4^ Overwhelmingly in low- and middle-income countries, especially in sub-Saharan Africa, short-acting methods by far predominate.

**Figure.**
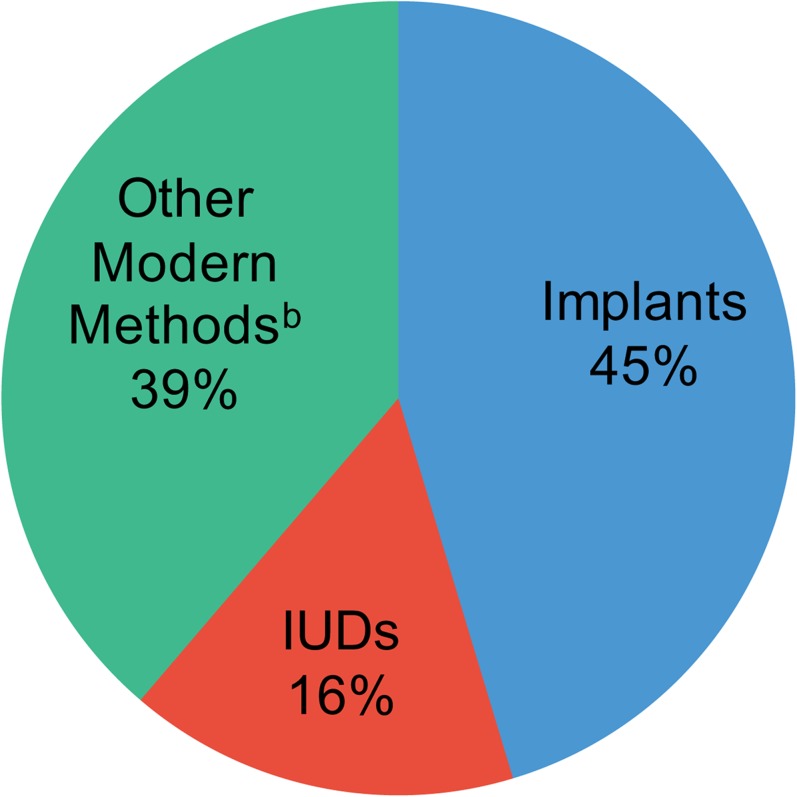
Method Mix Among New Modern Method Users at CARE-Supported Facilities in 5 Crisis-Affected Countries,^a^ July 2011–December 2013 ^a^ Chad, Democratic Republic of the Congo, Djibouti, Mali, and Pakistan. ^b^ Other modern methods included injectables, oral contraceptive pills, tubal ligation, and vasectomy.

When provided with a wide choice of methods, 61% of women in crisis settings selected LARCs.

Why this difference? It seems inescapable that short-acting methods predominate because they are by far the most available in most settings. Providing pills, condoms, and injectables is relatively easy, but it is not so easy to provide LARCs and permanent methods. Still, IUDs are widely used in countries such as Egypt and Viet Nam, and sterilization is highly used in India and Latin America. And we have seen that, with robust service delivery models such as mobile outreach and social franchising, use of implants is highly acceptable in Africa.^5^ So programs can successfully provide such methods.

Provision of implants through mobile outreach and social franchising models has been highly acceptable in Africa.

Granted, it may well be true that women in crisis-affected situations are more interested in preventing pregnancy for a longer time. However, provision of LARCs in the CARE program was actually constrained somewhat. In Pakistan, implants provision was impaired by difficulty in securing supply and gaining government approval for community health workers (Lady Health Visitors) to insert implants. In Djibouti, training to provide implants and IUDs was delayed. And availability of sterilization was limited overall. Moreover, bear in mind the CARE data are on method selection *for new clients*. Over time, the proportion and prevalence of LARC use among that same set of women will increase, because continuation of LARCs is much better than that of short-acting methods. Notably, the proportion of new clients selecting IUDs increased progressively over time in crisis settings. And good availability of permanent methods would likely shift the method mix even more.

This all presents a major opportunity. Providing a more balanced method mix, by improving access to long-acting methods, allows family planning programs to meet clients' individual needs much better. And long-acting methods generally enable more successful contraception than short-acting methods, with much lower failure rates and far better continuation rates. Thus, a more optimal method mix will prevent unintended pregnancy better and increase the health benefits of healthy timing and spacing of pregnancy.

The program mandate is clear—priority for well-executed service delivery models, such as mobile outreach, social franchising, and other approaches, for robust, quality provision of LARCs and permanent methods, in the context of choice among a broad range of methods.—*Global Health: Science and Practice*

## Notes

**First published online:** 2015 Feb 25
